# TcaR–ssDNA complex crystal structure reveals new DNA binding mechanism of the MarR family proteins

**DOI:** 10.1093/nar/gku128

**Published:** 2014-02-14

**Authors:** Yu-Ming Chang, Chun-Han Ho, Cammy K.-M. Chen, Manuel Maestre-Reyna, Masatoshi Weiting Chang-Chien, Andrew H.-J. Wang

**Affiliations:** ^1^Institute of Biological Chemistry, Academia Sinica, Taipei 11529, Taiwan, ^2^Institute of Biochemical Sciences, National Taiwan University, Taipei 106, Taiwan, ^3^Core Facilities for Protein Structural Analysis, Academia Sinica, Taipei 11529, Taiwan and ^4^Graduate Institute of Translational Medicine, College of Medical Science and Technology, Taipei Medical University, Taipei 110, Taiwan

## Abstract

The teicoplanin-associated locus regulator (TcaR) regulates gene expression of proteins on the intercellular adhesion (ica) locus involved in staphylococci poly-*N*-acetylglucosamine biosynthesis. The absence of TcaR increases poly-*N*-acetylglucosamine production and promotes biofilm formation. Until recently, the mechanism of multiple antibiotic resistance regulator family protein members, such as TcaR, was restricted to binding double-stranded DNA. However, we recently found that TcaR strongly interacts with single-stranded DNA, which is a new role for this family of proteins. In this study, we report *Staphylococcus epidermidis* TcaR–single-stranded DNA complex structures. Our model suggests that TcaR and single-stranded DNA form a 6_1_-symmetry polymer composed of TcaR dimers with single-stranded DNA that wraps outside the polymer and 12 nt per TcaR dimer. Single-stranded DNA binding to TcaR involves a large conformational change at the DNA binding lobe. Several point mutations involving the single-stranded DNA binding surface validate interactions between single-stranded DNA and TcaR. Our results extend the novel role of multiple antibiotic resistance regulator family proteins in staphylococci.

## INTRODUCTION

Staphylococci are among the most common bacterial pathogens and pose a major danger to human health. *Staphylococcus aureus* and *Staphylococcus epidermidis* are well-studied infectious agents responsible for the majority of both multiresistant and nosocomial infections from methicillin-resistant *S. aureus* (MRSA) and are a serious threat to public health (1). The transcription regulator teicoplanin-associated locus regulator (TcaR) is a member of the multiple antibiotic resistance regulator (MarR) family involved in teicoplanin and methicillin resistance in staphylococci (2). TcaR regulates *icaADBC* (3), *spa*, *sasF* (the first reported regulator for cell wall-anchored proteins) and *sarS* (4,5) transcription.

We previously described staphylococci MarR protein 3D structures in the apo form and in complex with salicylate, aminoglycoside and β-lactam antibiotics (6,7). Therein, we compared the apo MarR protein and MarR-antibiotic complexes, which indicated that the regulation mechanism involved a large conformational shift in the DNA binding lobe. Until recently, MarR family member DNA binding was restricted to double-stranded DNA (dsDNA) binding (8–10). However, we showed that TcaR interacted strongly and cooperatively with single-stranded DNA (ssDNA) (11), which suggests a novel TcaR function in regulating DNA replication and in resisting ssDNA phage invasion. However, a detailed mechanism on the interaction between TcaR and ssDNA has not been elucidated.

In this study, a crystal structure for TcaR in the ssDNA-bound form was solved to better understand how the interaction between TcaR with ssDNA regulates multiple TcaR functions. We compare the apo TcaR and TcaR–ssDNA complex structures, which indicates that the interaction mechanism involves a significant conformational change in the DNA binding lobe. Furthermore, TcaR expression was enhanced by viral ssDNA M13 transformation in *S. epidermidis*, which shows an interesting link between MarR proteins and ssDNA phage resistance. Taken together, these results provide an indepth analysis of the multiple TcaR functions in *S. epidermidis*.

## MATERIALS AND METHODS

### Cloning, protein expression and purification

TcaR gene expression and protein purification were performed in accordance with previously described methods (7,11). The biofilm-forming *S. epidermidis* strain ATCC 35984 (RP62A) was acquired from the Food Industry Research and Development Institute in Taiwan. The *tcaR* gene was directly amplified from the *S. epidermidis* RP62A genome through polymerase chain reaction (PCR). The PCR product encoding TcaR with an amino-terminal His_6_ tag was digested with NdeI and HindIII and subsequently cloned into the expression vector pET-21b(+) (Novagen). This construct was transferred into the *Escherichia coli* Arctic Express™ (DE3) RIL strain. DNA sequencing was performed to confirm the appropriate orientation. The His_6_-tagged wild-type protein was overexpressed in Difco Luria-Bertani (LB) broth with 50 mg/l ampicillin to a 600-nm optical density at 0.5–0.6 and then induced with 0.5 mM Isopropyl β-d-thiogalactopyranoside (IPTG). The cells were grown for 2 days at 13°C. The cells were then harvested through centrifugation at 12 000 *×g* for 30 min and disrupted using a Constant Cell Disruption System (CONSTANT SYSTEM Ltd, UK) with lysis buffer containing 20 mM Tris-HCl (pH 8.5), 400 mM NaCl and 10 mM imidazole. The homogenate was centrifuged at 27 000 *×g* for 30 min, and the cell-free extract was loaded onto an Ni^2+^-NTA column, which was previously equilibrated with lysis buffer. The column was washed with lysis buffer, and the His_6_-tagged TcaR was subsequently eluted through a linear imidazole gradient from 10 mM to 500 mM. The fractions with purified TcaR were collected and dialyzed three times for 6 h against 5 l of dialysis buffer (first, 20 mM sodium citrate, 10% glycerol, 300 mM NaCl and pH 4.5; second, 20 mM sodium citrate, 10% glycerol, 200 mM NaCl and pH 4.5; third, 20 mM sodium citrate, 10% glycerol, 100 mM NaCl and pH 4.5). The purified His_6_-tagged TcaR was finally concentrated using Amicon ultra-15 centrifugal filter units (3 kDa MWCO) (Millipore, MA, USA) and stored at −80°C. The molecular weight of the purified protein was verified using mass spectrometry, and the purity (>95%) was measured through SDS-PAGE.

### Electrophoretic mobility shift assays

The GC33 oligonucleotide probe used in the electrophoretic mobility shift assay (EMSA) experiments was purchased from MDBio Inc. (Taiwan) (11). The viral φX174 and M13 ssDNA were purchased from New England Biolabs (USA). Gel shift assays were performed by incubating ssDNA (12 μM final concentration) with different quantities of purified TcaR (2–24 μM final concentration) under binding conditions (20 mM Tris-HCl, pH 8.0, 150 mM KCl, 0.1 mM MgCl_2_, 0.05 mM ethylenediaminetetraacetic acid (EDTA), 12.5% glycerol, 0.1 mM dithiothreitol and 1 mg/ml bovine serum albumin) for 15 min at room temperature with gentle vortexing. After incubation, 15 μl of the reaction solution was mixed with 3 μl of the sample loading dye and subsequently electrophoresed on 6% polyacrylamide gels in 0.5×Tris-acetate-EDTA (TAE) buffer (20 mM Tris-acetate and 0.5 mM EDTA, pH 8.3) at 100 V for 30 min and visualized using SYBR Green I nucleic acid gel stain (Invitrogen).

### Crystallization and data collection

For crystallization, the TcaR concentration was adjusted to 18 mg/ml in 20 mM sodium citrate, pH 4.5, 10% glycerol, 100 mM NaCl and 5 mM dithiothreitol. In addition, the ssDNA probe 5′-CGCAGCGCGCAGCCCTA-3′, which was used for crystallization, was purchased from MDBio Inc. (Taiwan). Before crystallization, a 17-mer oligonucleotide was pre-incubated with a 1:1 molar ratio of TcaR (dimer) (200 μM final concentration) at room temperature for 15 min. The TcaR–ssDNA co-crystals were then generated in 40% ethylene glycol and 0.1 M Tris, pH 7.0. High-quality crystals continued to grow to full size for >30 days at room temperature. The X-ray diffraction data sets for the TcaR complexes were collected at SPring-8 (Hyogo, Japan), beamline BL44XU. The data were processed and scaled using the HKL2000 (12). The data collection statistics are summarized in Supplementary Table S1.

### Structure determination, model building and refinement

The TcaR–ssDNA complex structure was determined by molecular replacement using the native TcaR (3KP7) structure as the search model. The remaining portions of the model were manually built and further refined using the programs Xtalview (13) and *CNS* (14) with guidance from the (2F_o_–F_c_) sum difference maps. The model was manually adjusted using COOT (15) and refined with Phenix (16). The stereochemical quality was assessed using *MolProbity* (17,18). The illustrations were produced using PyMOL (DeLano Scientific, http://www.pymol.org) and Chimera (19).

### Real-time quantitative PCR data analysis

For the quantitative PCR experiments, an overnight culture was grown by inoculating 10 ml of Bacto™ tryptic soy broth (TSB; Difco, USA) medium with 100 μl of a frozen *S. epidermidis* stock (160 rpm, 37°C) (20,21). When the bacterial culture approached a 0.4 OD_600_, it was either grown with or without antibiotics or first transformed with virion M13 ssDNA and then grown with or without antibiotics for an additional 6 h. Every hour, the total RNA was extracted using the High Pure RNA Isolation kit (Roche) for one aliquot (1 ml) from each culture. The *A*_260 nm_/*A*_280 nm_ ratio (which was consistently >1.8) was measured to assess the nucleic acid purity. Reverse transcription was performed using 500 ng of total RNA, 100 pmol of anchored-oligo (dT)_18_ primer and 100 U of reverse transcriptase. Reverse transcription was assessed using the Transcriptor First Strand cDNA Synthesis kit (Roche). The reactions were treated as follows: the RNA sample was preheated for 10 min at 65°C; the reaction mixture was added on ice, heated for 1 h at 50°C, heated at 85°C for 5 min for enzyme denaturation and rapidly cooled to 4°C.

The reactions were quantified using a Light Cycler apparatus and the LightCycler® 480 Probes Master (Roche). Each reaction mixture contained a 1:10 dilution of cDNA (5 μl), 500 nM primer, 250 nM Taqman and quencher dye 6-carboxyfluorescein (6′-FAM) to the final volume of 20 μl. After 10 min at 95°C, 45 thermal cycles were performed (10 s at 95°C, 10 s at 55°C and 15 s at 72°C). The results were analyzed using the included software. Crossing points were established using the second derivative method. The TcaR transcription levels were measured relative to expression of an internal standard 16 S rRNA. The experiments were performed in triplicate and repeated three times independently, and the data were plotted as the mean ± SD.

### Phage studies

The TcaR protection assay was performed using *E. coli* as a host to examine the ability of TcaR to protect *E. coli* against M13 phage infection. An overnight culture with a single transformant was diluted 100-fold in a 5-ml LB broth and inoculated with phage M13 at a 0.01 multiplicity of infection and incubated at 37°C for 200 rpm. The growth (OD_600_) was measured as previously described (22). LB was supplemented with the following antibiotics: ampicillin 100 μg/ml and chloramphenicol 34 μg/ml. IPTG was added to a 0.1-mM final concentration when the OD_600_ reached ∼0.4. The experiments were performed at least in triplicate and plotted as the mean ± SD.

### Protein Data Bank accession codes

The atomic coordinates and structure factors for the TcaR–ssDNA complex were deposited in the wwPDB with the accession number 4KPD.

## RESULTS AND DISCUSSION

### Structure of the TcaR–ssDNA complex

**Figure 1.**
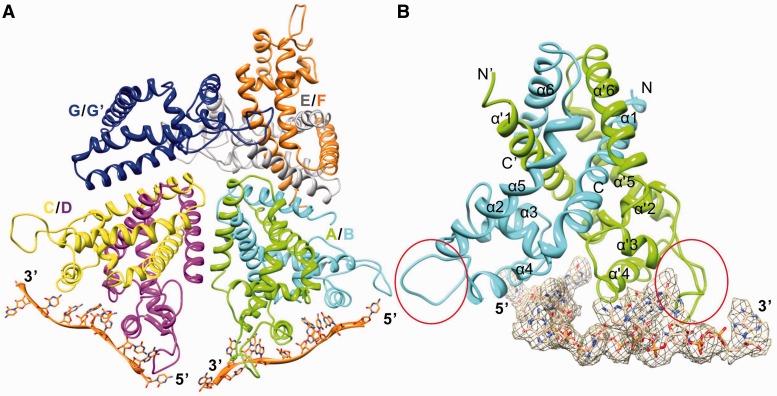
The overall TcaR–ssDNA complex structure. (**A**) The crystal includes three TcaR dimers and one monomer per asymmetric unit. The TcaR dimers A/B (colored in green/cyan) and C/D (colored in yellow/magenta) interact with ssDNA, whereas the dimer E/F (colored in gray/orange) does not. (**B**) The final 2Fo-Fc omit density map with the nucleic acids contoured at a 1δ level is shown in gray mesh. The β-wing region is labeled with red ovals.

To understand how the TcaR–ssDNA interaction regulates multiple TcaR functions, we report the crystal structure for TcaR in the ssDNA-bound form at a 3.6-Å resolution (Supplementary Table S1). The structure was solved through molecular replacement using the initial phases derived from our native structure (Figure 1A) (6). *P*3_2_21 TcaR crystals were grown with a 17-mer GC-rich ssDNA. The electron density map enabled us to build the nucleic acids (Figure 1B). Each asymmetric unit contained seven TcaR and two ssDNA molecules with two TcaR dimers that interacted with one ssDNA chain, one apo TcaR dimer and one apo TcaR monomer, respectively (Figure 1A). Although we only built 10–11 nt for each TcaR–ssDNA complex, the full-length (17-mer) ssDNA was confirmed by dissolving the complex crystals (TcaR–ssDNA) in water after washing them a few times in the reservoir solution; we then analyzed the ssDNA oligomers using agarose gel electrophoresis.

The TcaR complex structure reveals the full protein shape, including the β-wing region (Figure 1B), which suggests that ssDNA stabilizes this motif. This observation is consistent with our structure of full-length TcaR (4EJU) (7). Furthermore, the TcaR dimer in the complex differs from our previous TcaR–dsDNA complex model (6) (Supplementary Figure S1) with a 3.4-Å root mean square deviation using 288 Cα atoms. A major conformational difference was observed for DNA binding wHTH motifs. In the ssDNA complex, the motifs twist with respect to each other, which produces a sheared orientation and shortens the distance between the α3/3′ helices’ C-termini by 11.8 Å compared with the dsDNA complex (the Cα-Cα distance between Lys A60 and Lys B60). Moreover, upon ssDNA complex formation, the DNA binding wing was displaced by as much as 18 Å (measured from the Asn 89 Cα) from its likely position in the dsDNA minor groove. This motion is asymmetric and might generate unfavorable TcaR–dsDNA interactions in the ssDNA complex. Although previous experiments indicate that the ssDNA binding site and dsDNA binding site are proximal (11), we suggest different modes of interaction in the ssDNA–TcaR and dsDNA–TcaR complexes.

### Interactions between TcaR and ssDNA

**Figure 2.**
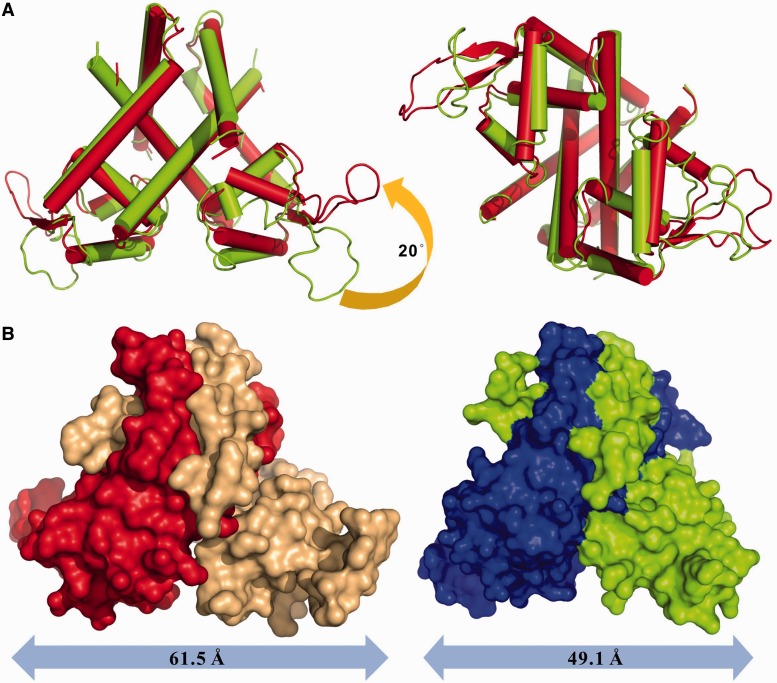
Structural comparison of the TcaR–ssDNA complex. (**A**) (Left) The TcaR apo (red) and ssDNA complex (green) structures are superimposed in cartoon mode. The TcaR complexes show significant conformational changes at the wHTH domain. (Right) The TcaR complexes are shown after an ∼90° rotation along the horizontal axis relative to the plane of the paper. (**B**) (Left) The apo TcaR dimer surfaces are colored red for Chain A and brown for Chain B. (Right) The TcaR–ssDNA complex surfaces are colored green for Chain A and blue for Chain B.

By superimposing the TcaR subunits bound to ssDNA from the *P*3_2_21 crystals and the apo TcaR dimer from the *P*6_1_ crystal forms (4EJU), we show an ∼1.7-Å root mean square deviation (Figure 2A). Such changes are primarily confined to local wHTH motif rearrangements. To bind the ssDNA oligomers, the DNA-binding wHTH motif of one apo TcaR dimer subunit rotates by ∼20°, and the wing tips translocate ∼21.4 Å (measured from the Asn 89 Cα, where prime indicates residues from the second subunit). Thus, the ssDNA-bound TcaR recognition helices move closer together, and the intersubunit tip-to-tip distances decrease from 61.5 Å to 49.1 Å (measured from the Asn 89 Cα). In addition, the TcaR–ssDNA complex winged wHTH motif shifts down compared with the apo TcaR. The wHTH motifs twist in relation to each other to produce a sheared orientation, wherein an interaction with the target DNA is highly unlikely. Furthermore, ssDNA binding reduces the winged DNA-binding domain flexibility and decreases the distance between the wHTH motifs. Although it is challenging to distinguish whether the conformational changes are ssDNA oligomer-induced or caused by crystal-packing differences, we observed important structural differences and a notable plasticity in TcaR. The observations indicate that steric occlusion at the ssDNA–TcaR interface may inhibit DNA binding. Comparing the surface conformation between the apo TcaR and TcaR–ssDNA complex also highlights an asymmetric structural change (Figure 2B), which is similar to that observed in the TcaR–antibiotic complexes (6).

In total, 18 amino acids from the TcaR dimer form 22 DNA contacts over a 21-nt span (Supplementary Figure S2A–C and Supplementary Table S2). Among the interacting residues, Arg 70, Lys 74 and Arg 93 are strongly conserved in MarR family proteins. We previously demonstrated that a TcaR quadruple mutant (R70A/K74A/R93A/K95A) did not interact with 33-mer ssDNA oligomers (11). Here, we also show that the quadruple mutant does not bind φX174-ssDNA (Supplementary Figure S3A), which confirms that these residues are crucial for TcaR–ssDNA interactions.

### TcaR–ssDNA polymer model

**Figure 3.**
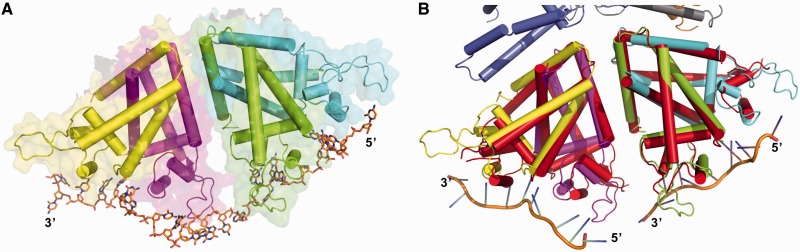
(**A**) The TcaR-24-nt-ssDNA complex model. The model was constructed based on the crystal structure of the complex formed by the TcaR dimers A/B (colored in green/cyan) and C/D (color in yellow/magenta). (**B**) Structural comparison of the dimer–dimer interactions in the TcaR apo (red) and ssDNA complex structures.

**Figure 4.**
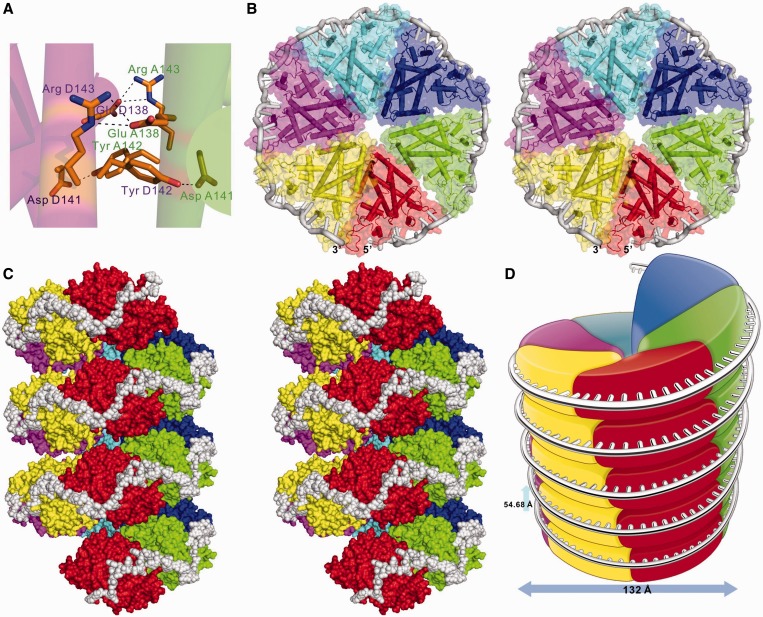
Model of the TcaR-long-chain–ssDNA complex. (**A**) Detailed interactions between TcaR dimers A/B (colored in green/cyan) and C/D (colored in yellow/magenta). (**B** and **C**) Stereo view of the combined model for the ssDNA location in the TcaR filament, as shown along the helical *c* axis in the space group *P*6_1_ (b: bottom view; c: side view). The counterclockwise extended TcaR filament is right-handed with six TcaR molecules per turn and a 0.76 -Å rise per nucleotide. The TcaR filament sequence is red-green-blue-cyan-magenta-yellow. (**D**) Proposed model of the TcaR-long-chain–ssDNA complex. The complex has a 54.68 -Å helical pitch with a 132 -Å diameter. Each TcaR dimer interacts with 12 nt.

In the TcaR–ssDNA complex, we observed that the dimers A/B and C/D (colored in green/cyan and magenta/yellow) interacted with 11-mer and 10-mer ssDNA oligomers, respectively. The A/B-DNA 3′-end and C/D-DNA 5′-end are 21.3 Å apart, which facilitated modeling of 3 or 4 connected oligonucleotides. In addition, we used the TcaR A/B-ssDNA and C/D-ssDNA heterotrimeric complexes as templates to construct an extended TcaR–ssDNA complex model (Figure 3A). In this model, two TcaR dimers interact with 24-nt-long ssDNA oligomers, which produce a 60° rotation in the single-strand backbone. The winged-loop region that connects the TcaR helices α4 and α5 helped stabilize the ssDNA molecule. When we superimposed the TcaR–ssDNA complex and apo TcaR dimer from the *P*6_1_ crystal (3kp7), we observed identical interactions between the dimers A/B and C/D (Figures 3B and 4A) with a salt bridge between Arg 143 and Glu 138 and a hydrogen bond between the Asp 141 carboxylate group and Tyr 142 phenolic OH. The aforementioned residues might be responsible for ultrastructural interactions, which generate cooperative binding between TcaR and long ssDNA fragments. Our previous studies found that other MarR proteins including *S. aureus* SAR2349 and *E. coli* MarR appeared to have no interactions with ssDNA in EMSA and surface plasmon resonance (SPR) analysis (11). Interestingly, Glu 138, Asp 141, Tyr 142 and Arg 143 are unique to TcaR and not conserved among the other MarR proteins (Supplementary Figure S4), which might explain why only TcaR has been reported to bind to ssDNA. To confirm this hypothesis, the triple mutant D141A/Y142A/R143A was designed. As shown in Supplementary Figure S3B, compared with a native TcaR protein, the triple mutant viral ssDNA binding affinity was lower. The result shows that the dimer–dimer interacting residues are important for binding longer ssDNA fragments.

The TcaR–ssDNA complex crystal structure (Figure 1A) was used as a template to model the TcaR–viral ssDNA complex (Figure 4B–D). In this model, the TcaR proteins form a helical filament through dimer–dimer interactions with six TcaR molecules per turn (54.68 Å in helical pitch and 132 Å in diameter) and 0.76 Å rise per nucleotide, which correlates with the *P*6_1_ 6-fold axis. One TcaR dimer interacted with a 12-nt-long ssDNA oligomer, which produced a 60° rotation in the single-strand backbone. Therefore, TcaR protein binding to a long-chain ssDNA molecule forms a highly condensed structure, and the DNA is compacted by ∼5-fold compared with its length in the extended double helical form. Our model slightly differs from a previous electron microscopy (EM) analysis (11). We suggest that the conditions and molar ratio between TcaR and the φX174 ssDNA may not have been the most suitable in previous EM experiments. The TcaR protein concentration may have been excessive and produced aggregation in certain portions of the TcaR-φX174 complex. Moreover, we were also unable to generate an image with high resolution for reconstruction and comparison with our TcaR–ssDNA complex. We are currently applying an EM technique to image the TcaR-φX174 and TcaR-M13 complexes specifically labeled with 1.8 nm of nano-gold. The 3D reconstruction of the gold-bound complexes will be used to discern the specific location of TcaR on the viral ssDNA. This study is in progress.

### Biological implication

**Figure 5.**
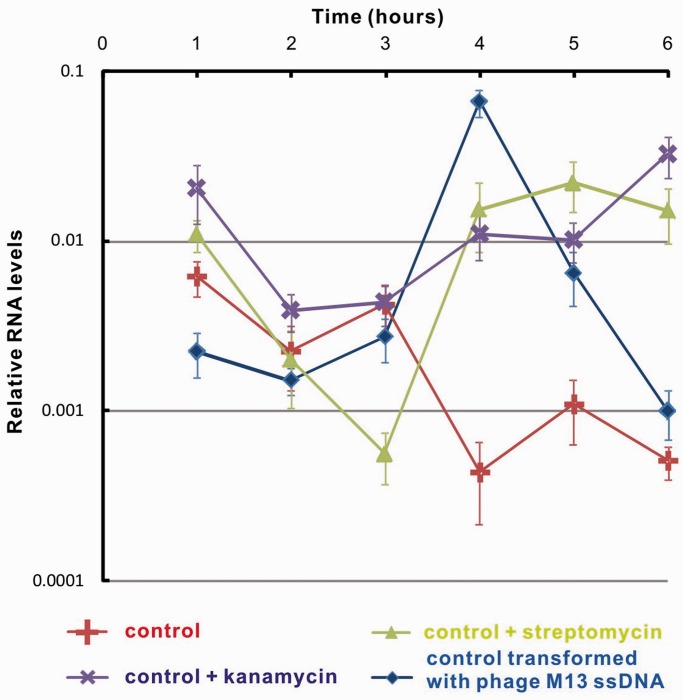
RT-PCR analysis of TcaR transcripts in *S. epidermidis* RP62A under different growth conditions. Relative levels of TcaR expressions were calculated compared with those of the control, 16S rRNA. The mean ± SD for the quantitative RT-PCR data is shown (*n *= 3).

Previously, we investigated an anti-phage system for the ssDNA phage wherein TcaR expression inhibits phage M13 and φX174 replication (11). Here, we also show that inducing the TcaR protein in *E. coli* increased the host resistance to ssDNA phage M13 infection (Supplementary Figure S5). Although the non-lytic M13 virus decelerates *E. coli* growth, an induced TcaR expression inhibits M13 phage replication and rescues the host from viral infection. To further confirm and clarify the relationship between the TcaR protein and ssDNA phage resistance in *S. epidermidis*, quantitative reverse transcriptase polymerase chain reaction (RT-PCR) was used to measure *TcaR* mRNA transcript accumulation. As a control, the housekeeping gene 16S rRNA was used to normalize the expression levels (20,21). The quantity of *TcaR* mRNA in *S. epidermidis* increased both after viral M13 ssDNA transformation and antibiotic treatment (Figure 5), which is consistent with previous findings on the effects of an antibiotic treatment on *MarR* genes (23–25). In contrast to the constantly upregulated TcaR expression following an antibiotic treatment, rapid TcaR gene activation after M13 ssDNA transformation was followed by markedly reduced transcription. TcaR transcription activity was upregulated 53.3-fold by the fourth hour compared with the first hour; interestingly, the activity was quickly downregulated by a factor of 78.1 on the sixth hour compared with the fourth hour, which indicates that this regulation mechanism is highly dynamic and can rapidly adapt for survival and growth in divergent situations. Such a mechanism differs from the types of microbial antibiotic resistance previously reported (26,27). Although the exact mechanism is not well understood, we suggest that a viral infection could trigger TcaR expression in staphylococci in a short time, and TcaR exhibits a defensive response by inhibiting the subsequent phage DNA replication. After TcaR completes its viral ssDNA replication blocking function, its transcription activity is also downregulated to a normal condition by another unknown mechanism to maintain typical antibiotic resistance in staphylococci.

## CONCLUSION

Until now, most studies on MarR–nucleic acid interactions have concentrated on dsDNA. The MarR family protein–ssDNA interaction appears to modulate the multiple TcaR functions but still requires elucidation. Improved understanding of such interactions will not only aid in understanding numerous biological mechanisms, but it may also provide a basis for designing new therapies against staphylococcal infection. In this study, we report the first structures of a TcaR–ssDNA complex and show a detailed interaction mechanism for TcaR and ssDNA. We further clarify the role of the TcaR–ssDNA interaction using real-time quantitative PCR data analyses. This study is generally interesting because it displays the remarkably broad spectrum of nucleic acid-binding modes in multifunctional MarR proteins. This finding expands our understanding of MarR proteins in pathogens and suggests strategies to develop new therapies against them.

## ACCESSION NUMBERS

The atomic coordinates and structural factors for TcaR complexed with ssDNA (4KDP) have been deposited in the RCSB Protein Data Bank.

## Supplementary Material

Supplementary Data
