# Supplementary material for: TcaR–ssDNA complex crystal structure reveals new DNA binding mechanism of the MarR family proteins
Source: Nucleic Acids Res. 2014 Feb 14;42(8):5314–21. doi: 10.1093/nar/gku128 (PMC4005659; doi:10.1093/nar/gku128)
Supplement: Supplementary Data [file supp_42_8_5314__index.html]

TcaR–ssDNA complex crystal structure reveals new DNA binding mechanism of the MarR family proteins — TcaR–ssDNA complex crystal structure reveals new DNA binding mechanism of the MarR family proteins — Supplementary Data 

# TcaR–ssDNA complex crystal structure reveals new DNA binding mechanism of the MarR family proteins

## Supplementary Data

files

**Files in this Data Supplement:**

- Supplementary Data - doc file
