# Supplementary material for: TcaR–ssDNA complex crystal structure reveals new DNA binding mechanism of the MarR family proteins
Source: Nucleic Acids Res. 2014 Feb 14;42(8):5314–21. doi: 10.1093/nar/gku128 (PMC4005659; doi:10.1093/nar/gku128)
Supplement: Supplementary Data [file supp_gku128_nar-02546-m-2013-File007.doc]

**TcaR-ssDNA Complex Crystal Structure Reveals New DNA Binding Mechanism of the MarR Family Proteins**

Yu-Ming Chang, Chun-Han Ho, Cammy K.-M. Chen, Manuel Maestre-Reyna, Masatoshi Weiting Chang-Chien and Andrew H.-J. Wang*

**Supplementary Data**

**Table S1.** Data collection and refinement statistics for the TcaR-ssDNA crystal.

| Names | TcaR-ssDNA complex |
| --- | --- |
| PDB number | 4KDP |
| Data Collection | |
| Space group | *P*3_2_21 |
| Resolution (Å)*^a^* | 30-3.6 |
|  | (3.73 –3.6) |
| Unit Cell Dimensions | |
| *a* (Å) | 91.90 |
| *b* (Å) | 91.90 |
| *c* (Å) | 261.53 |
| No. of reflections | |
| Observed | 46258(4422) |
| Unique | 15179(1474) |
| Completeness (%) | 98.8(99.7) |
| *R*_merge_ (%) | 5.2 (52.6) |
| I/δ(I) | 18.2(2.1) |
| Refinement | |
| No. of reflections | 14084(1245) |
| *R*_work_ (95% data) | 0.274 |
| *R*_free_ (5% data) | 0.285 |
| Geometry deviations | |
| Bond lengths (Å) | 0.020 |
| Bond angles (°) | 2.0 |
| No. of all protein atoms | 8512 |
| No. of all nucleic acid atoms | 442 |
| No. of water molecules | 67 |
| Ramachandran plot (%) | |
| Most favored | 75.4 |
| Additionally allowed | 21.3 |
| Generously allowed | 2.9 |
| Disallowed | 0.5 |

^a^ Values in the parenthesis are the highest resolution shells.

**Table S2. Protein-DNA interactions between TcaR and ssDNA.**

| TcaR | ssDNA |
| --- | --- |
| Lys B65 | Gua 2 |
| Ser A37 | Gua 2 |
| Lys A74 | Gua 2 |
| Lys A74 | Gua 2 |
| Arg B70 | Gua 2 |
| Arg B70 | Cyt 3 |
| Ser B37 | Ade 4 |
| Arg B71 | Ade 4 |
| Arg B71 | Ade 4 |
| Lys B74 | Gua 5 |
| Lys A65 | Cyt 6 |
| Gln A92 | Gua 7 |
| Arg A93 | Ade 11 |
| Arg A93 | Ade 11 |
|  |  |
| Lys D65 | Cyt 3 |
| Asn D64 | Cyt 5 |
| Arg D70 | Ade 6 |
| Arg D70 | Ade 6 |
| Ser D37 | Ade 6 |
| Arg D71 | Ade 6 |
| Lys D74 | Ade 6 |
| Lys C65 | Cyt 8 |

**
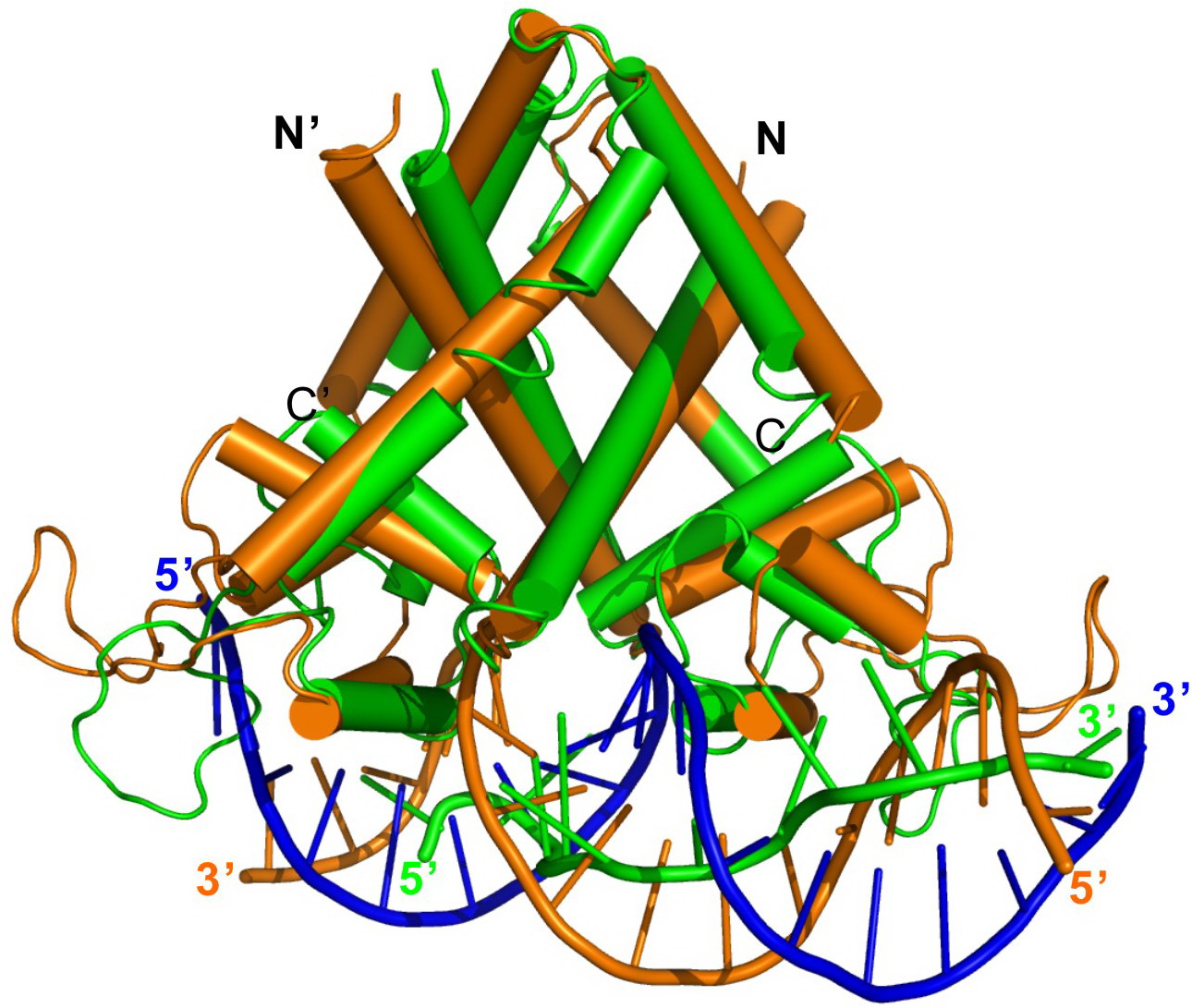
**

**Figure S1**

**
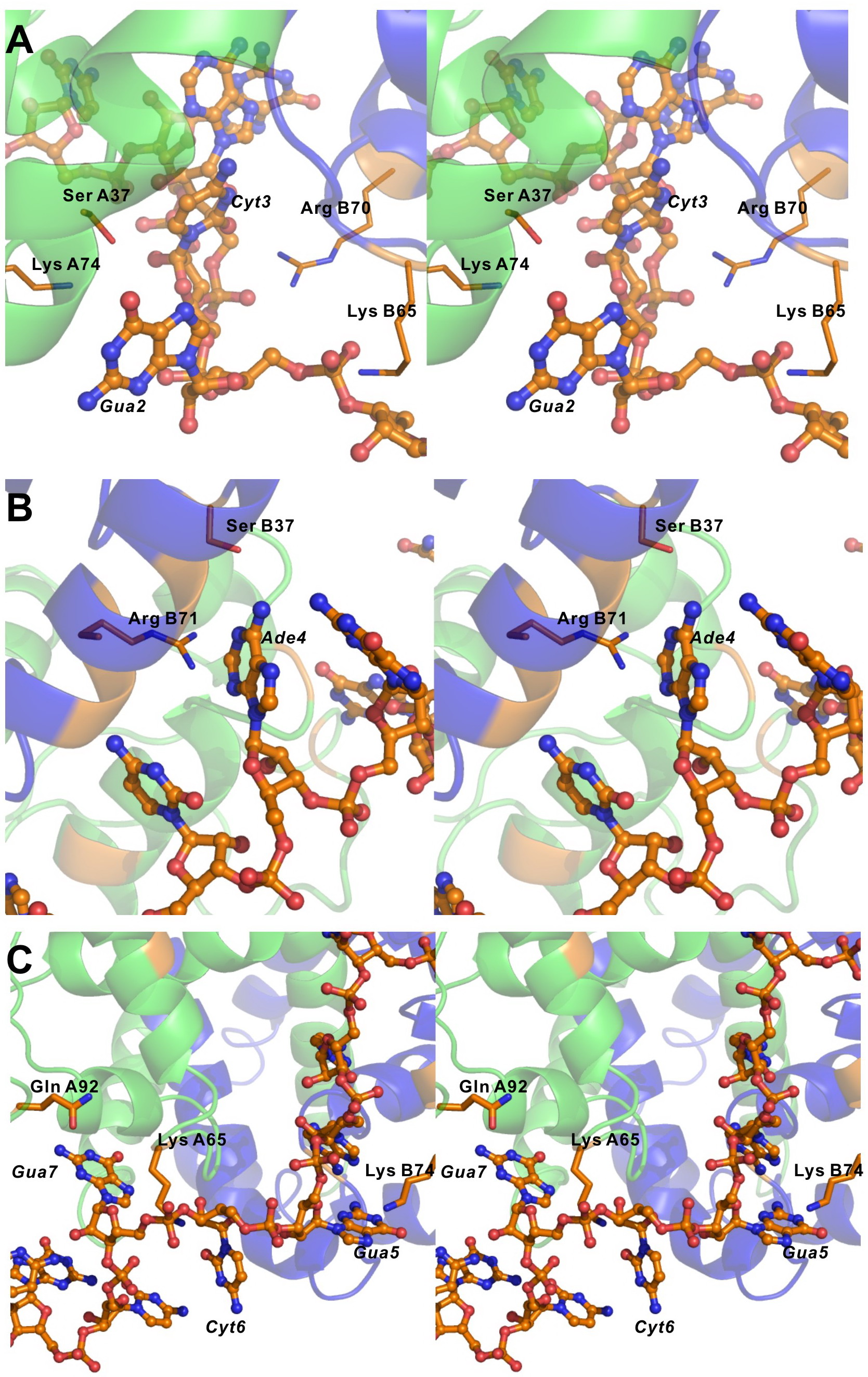
**

**Figure S2**

**
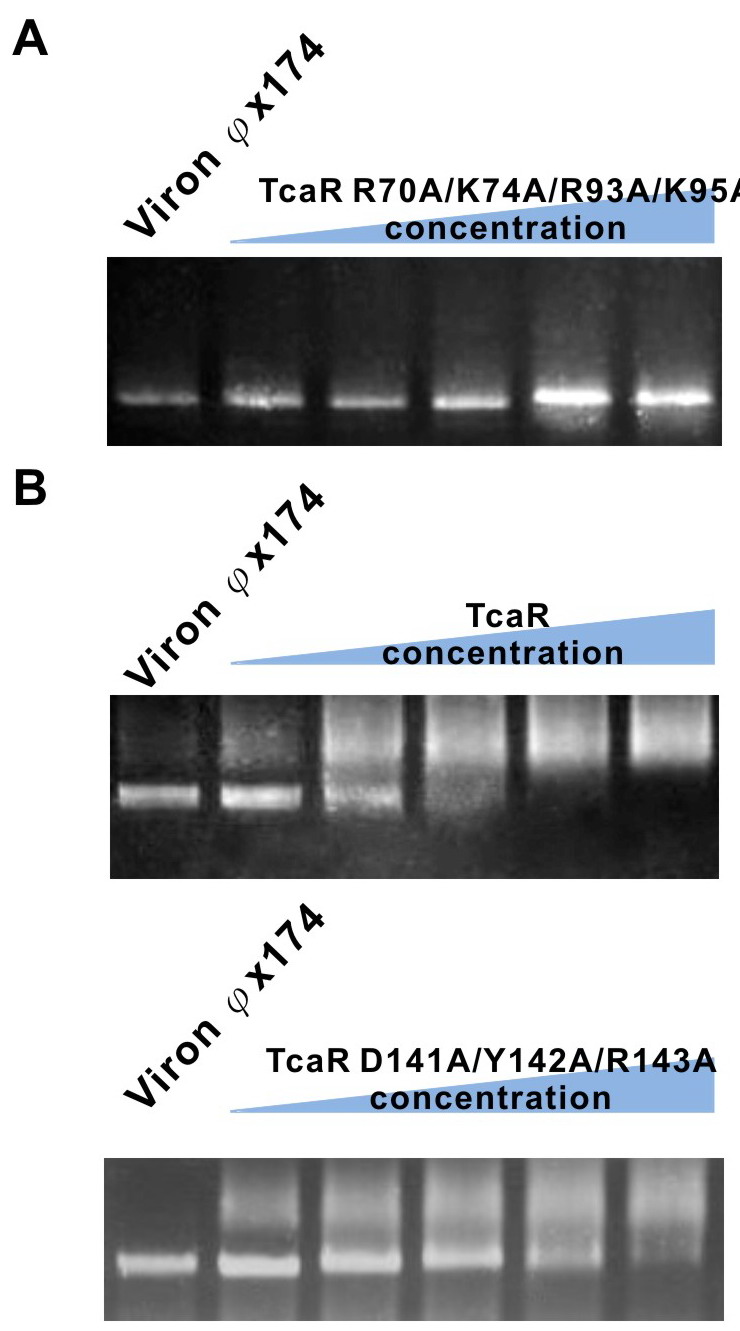
**

**Figure S3**

**
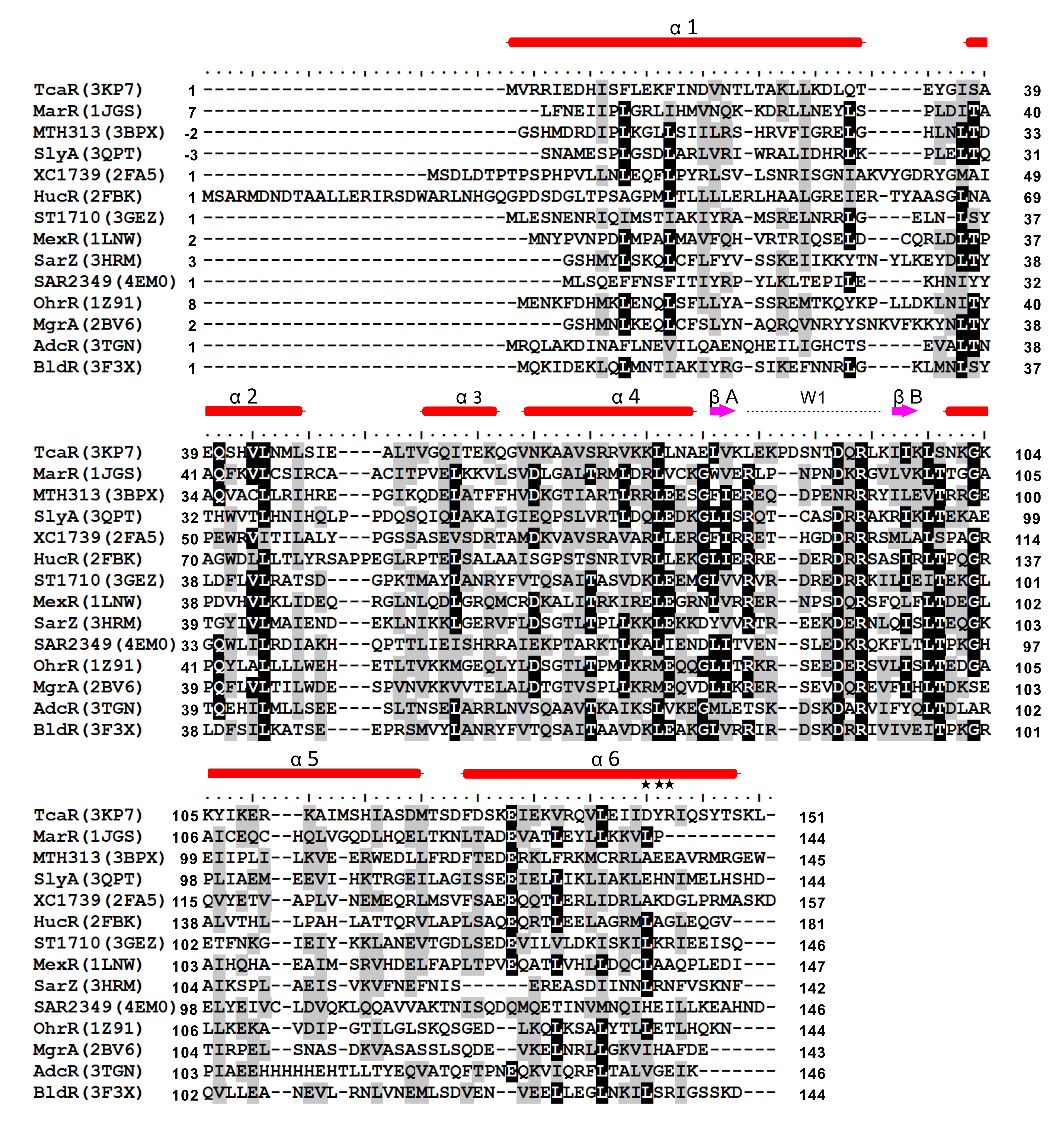
**

**Figure S4**

**
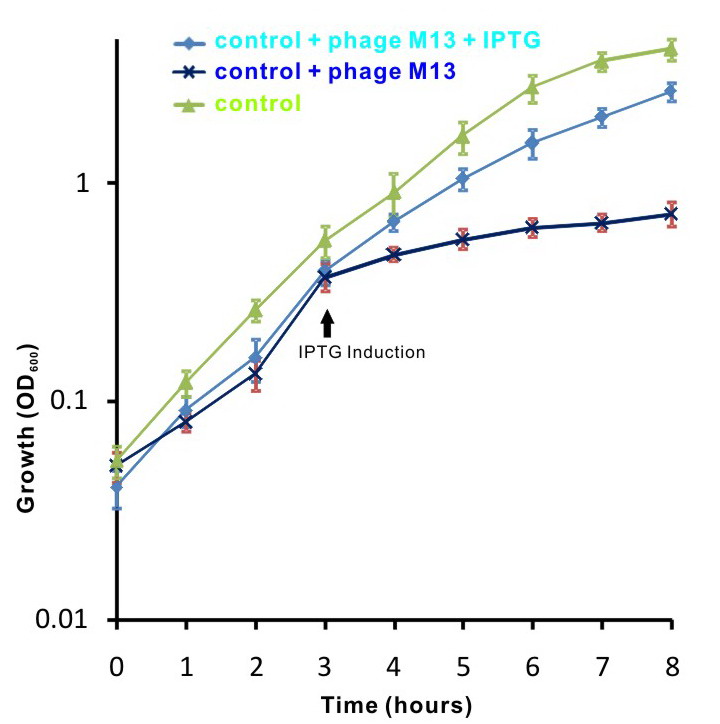
**

**Figure S5**

**Figures Legends**

**Figure S1. Structural comparison of the TcaR-ssDNA complex and TcaR-dsDNA model.** The protein complexes are shown as a ribbon diagram with TcaR-ssDNA complex in green and TcaR-dsDNA model in orange**.**

**Figure S2. (A-C)** Stereo views of the critical TcaR dimer A/B-ssDNA interface in the complex. The residues of the nucleic acids and proteins are shown using line, or stick models, respectively.

**Figure S3. Analyses for TcaR-viral ssDNA complex.** (**A**) EMSA analysis of the binding of TcaR quadruple mutant (R70A/K74A/R93A/K95A) to Viral φX174 ssDNA. Viral φX174 ssDNA (12 μM nucleotides) was incubated with an increasing concentration of TcaR mutant at 30 ℃ for 15 min. After incubation, 15 μl of the reaction solution was mixed with 3 μl of the sample loading dye and subsequently loaded onto a 0.8 % agarose gel and electrophoresed in 0.5 X Tris-acetate-EDTA  (TAE) buffer (20 mM Tris-acetate and 0.5 mM EDTA, pH 8.3) at 100 V for 30 min and visualized using SYBR Green I nucleic acid gel stain (Invitrogen). (**B**) EMSA analysis of the binding of native TcaR and triple mutant D141A/Y142A/R143A to viral φX174 ssDNA. Viral φX174 ssDNA (12 μM nucleotides) was incubated with an increasing concentration of TcaR proteins at 30 ℃ for 15 min. The reaction was analyzed by the same procedure as described in the legend to Fig. S3*a*.

**Figure S4. Sequence alignment of TcaR with other MarR proteins.** Amino acids that are conserved among the 14 sequences are shaded black, and similar residues are shaded gray. In addition, the residues marked with an “asterisk sign” form dimer-dimer interactions. The cylinders, arrows and dotted line represent α-helices, β-strains and the wing, respectively.

**Figure S5.**  **Protection of *E. coli* against phage M13 infection by the expression of TcaR.** Growth rate (OD_600_) of *E. coli* was measured before and after induction of TcaR gene; See Materials and methods for details. Mean ± SD of measurement data are shown (*n* ＝ 3).
